# Antiviral immunity is impaired in COPD patients with frequent exacerbations

**DOI:** 10.1152/ajplung.00253.2019

**Published:** 2019-09-12

**Authors:** Aran Singanayagam, Su-Ling Loo, Maria Calderazzo, Lydia J. Finney, Maria-Belen Trujillo Torralbo, Eteri Bakhsoliani, Jason Girkin, Punnam Veerati, Prabuddha S. Pathinayake, Kristy S. Nichol, Andrew Reid, Joseph Footitt, Peter A. B. Wark, Christopher L. Grainge, Sebastian L. Johnston, Nathan W. Bartlett, Patrick Mallia

**Affiliations:** ^1^National Heart and Lung Institute, Imperial College London, London, United Kingdom; ^2^Faculty of Health and Medicine and Priority Research Centre for Healthy Lungs, Hunter Medical Research Institute and University of Newcastle, Newcastle, New South Wales, Australia; ^3^Department of Respiratory and Sleep Medicine, John Hunter Hospital and Faculty of Health and Medicine and Priority Research Centre for Healthy Lungs, Hunter Medical Research Institute and University of Newcastle, Newcastle, New South Wales, Australia

**Keywords:** COPD, innate immunity, viral infection

## Abstract

Patients with frequent exacerbations represent a chronic obstructive pulmonary disease (COPD) subgroup requiring better treatment options. The aim of this study was to determine the innate immune mechanisms that underlie susceptibility to frequent exacerbations in COPD. We measured sputum expression of immune mediators and bacterial loads in samples from patients with COPD at stable state and during virus-associated exacerbations. In vitro immune responses to rhinovirus infection in differentiated primary bronchial epithelial cells (BECs) sampled from patients with COPD were additionally evaluated. Patients were stratified as frequent exacerbators (≥2 exacerbations in the preceding year) or infrequent exacerbators (<2 exacerbations in the preceding year) with comparisons made between these groups. Frequent exacerbators had reduced sputum cell mRNA expression of the antiviral immune mediators type I and III interferons and reduced interferon-stimulated gene (ISG) expression when clinically stable and during virus-associated exacerbation. A role for epithelial cell-intrinsic innate immune dysregulation was identified: induction of interferons and ISGs during in vitro rhinovirus (RV) infection was also impaired in differentiated BECs from frequent exacerbators. Frequent exacerbators additionally had increased sputum bacterial loads at 2 wk following virus-associated exacerbation onset. These data implicate deficient airway innate immunity involving epithelial cells in the increased propensity to exacerbations observed in some patients with COPD. Therapeutic approaches to boost innate antimicrobial immunity in the lung could be a viable strategy for prevention and treatment of frequent exacerbations.

## INTRODUCTION

Chronic obstructive pulmonary disease (COPD) is an inflammatory airway disorder in which acute exacerbations represent a major complication. Acute exacerbations are a substantial cause of morbidity and mortality (9, 31, 34) and preventing exacerbations remains a major unmet need. There has been increasing interest in a recently identified subgroup of patients with COPD, who are at risk of exacerbations, defined as the “frequent exacerbator phenotype”. (19) Patients with frequent exacerbations have worse clinical outcomes, including increased morbidity (31), accelerated lung function decline (9), and greater mortality (36), suggesting that this group requires special consideration. To date, the underlying mechanisms that predispose such patients to more frequent exacerbations have not been elucidated. It is plausible that abnormalities in host immunity could underlie an increased propensity to exacerbations.

Viruses are a major etiological trigger for exacerbations (28, 30), and data exist to suggest that COPD may be associated with deficient antiviral immunity (17, 22). However, not all studies have shown this abnormality (1, 20), suggesting that it may vary according to disease phenotype. Frequent exacerbators could represent one subgroup in whom defective antiviral immunity is more prominent. Both experimental and naturally occurring exacerbation studies also confirm that an initial virus infection can precipitate secondary bacterial infection in COPD (13, 21). Although bacterial colonization at a stable state has been shown to be associated with increased exacerbation frequency (26), propensity to develop secondary bacterial infection during virus infections in frequent versus infrequent exacerbators has not previously been studied.

Here, using analysis of sputum samples from a cohort of patients monitored prospectively in the community, in combination with in vitro experiments in bronchial epithelial cells (BECs) sampled from patients with COPD and differentiated at the air‐liquid interface (ALI), we show that innate antiviral immunity is impaired in frequent exacerbators. These mechanisms may underlie the increased propensity to exacerbation observed in some patients.

## METHODS

### 

#### St. Mary’s Hospital naturally occurring COPD exacerbation cohort.

A cohort of 40 COPD subjects was recruited for a longitudinal study carried out at St. Mary’s Hospital (London, UK) between June 2011 to December 2013 to investigate the pathogenesis of naturally occurring COPD exacerbations. The study protocol was approved by the East London Research Ethics Committee (study number 11/LO/0229), and all subjects gave informed written consent. The subjects all had a clinical diagnosis of COPD that was confirmed with spirometry. All subjects had an initial visit at baseline when clinically stable (infection-free without antibiotic treatment or treatment with oral corticosteroids for at least 8 wk) for clinical assessment, spirometry [forced expiratory volume in 1 s (FEV_1_), forced vital capacity (FVC), and peak expiratory flow (PEF)] and clinical sample collection, including spontaneous or induced sputum, taken and processed, as previously described (22, 27). At the baseline visit, all subjects were asked about the number of exacerbations experienced in the previous year and were divided into two groups: frequent exacerbators (≥2 exacerbations in preceding year) and infrequent exacerbators (0–1 exacerbations in the previous year), as previously described (38). Subjects then had repeat visits at three monthly intervals when clinically stable and were followed up for a minimum of 6 mo. Subjects reported to the study team when they developed symptoms of an upper respiratory tract infection or an increase in any of the symptoms of dyspnea, cough, sputum volume, or purulence. Exacerbation was defined using the East London cohort criteria (9). Subjects were seen within 48 h of onset of their symptoms for collection of samples, and repeat visits were carried out at 2 wk after the initial exacerbation visit. Samples from this cohort have been used in a previous study investigating the role of airway glucose in COPD (23).

#### RV infection of air‐liquid interface differentiated bronchial epithelial cells from patients with COPD.

Primary BECs obtained bronchoscopically from patients with COPD [Global Initiative for Obstructive Lung Disease (GOLD) Stage II or III] were grown until confluence and differentiated at the ALI, as previously described (14, 15, 33). All subjects included gave written informed consent, and the study protocol was approved by the Hunter New England Human Research Ethics Committee (05/08/10/3.09). Primary cells were grown in complete bronchial epithelial cell growth medium (BEGM) (Lonza) with growth factor supplements in submerged monolayer culture and then seeded at 2 × 10^5^ cells/mL in Transwell plates until confluent. RV-A1 infection [multiplicity of infection (MOI) 0.1] was applied to the apical surface for 2 h in 250 μl bronchial epithelial basal medium (BEBM) minimal at 35°C. Following this, infection media were replaced with 500 μl fresh BEBM minimal for the remainder of the experiment. Samples were collected at 72 h postinfection with apical media removed and stored for protein expression analyses. Type III IFN proteins were measured using a custom-designed LEGENDplex kit (BioLegend). IFN-β was measured using the Verikine ELISA (PBL). Half of the Transwell membrane was also carefully cut from the inserts and collected into RLT buffer (Qiagen) containing 1% 2-mercaptoethanol for downstream molecular analyses by RT-quantitative PCR. The remaining Transwell membrane was fixed in 10% neutral-buffered formalin for 24 h for histological cross sections to confirm differentiation (33).

#### Measurement of sputum proteins.

The MesoScale Discovery (MSD) platform (Maryland, USA) was used to measure inflammatory mediators IL-1β, IL-6, TNF, and CXCL8/IL-8 in sputum supernatants, according to manufacturers’ instructions, as published previously (10). Protein levels of CXCL10/IP-10 and CCL5/RANTES were measured using duoset ELISA kits (R&D Systems), according to the manufacturer’s instructions. Protein levels of all antimicrobial peptides were assayed using commercial ELISA assay kits, as previously described (3, 21). The sources of the individual ELISAs were as follows: elafin (R&D Systems), lactoferrin (Cambridge Bioscience), secretory leucocyte protease inhibitor (SLPI; R&D Systems), and surfactant protein-D (R&D Systems).

#### RNA extraction, cDNA synthesis, and quantitative PCR.

Total RNA was extracted from sputum cell pellets or bronchial epithelial cell lysates stored in RLT buffer (RNeasy kit; Qiagen), and 2 µg was used for cDNA synthesis (Omniscript RT kit). Quantitative PCR was carried out using previously described specific primers and probes for each gene of interest (35) and normalized to 18S rRNA. Reactions were analyzed using ABI 7500 Fast Realtime PCR system (Applied Biosystems).

#### Virus detection, DNA extraction, and bacterial 16S quantitative PCR.

DNA extraction from total sputum was performed using the FastDNA spin kit for soil (MP Biomedicals, Santa Ana, CA), as per the manufacturer’s instructions. Bead-beating was performed for two cycles of 30 s at 6,800 rpm (Precellys, Bertin Technologies, Montigny-le-Bretonneux, France). Total 16S bacterial loads were measured using quantitative PCR, as previously described (6). Viruses were detected as described previously (21).

#### Statistical analyses.

Comparisons of sputum mRNA expression and protein concentrations between frequent and infrequent exacerbators were analyzed using the Mann-Whitney *U* test. For in vitro experiments, baseline versus RV-induced expression was analyzed using ratio paired *t*-test. Mann Whitney *U* test was used to compare RV induction of mRNA or proteins between frequent and infrequent exacerbators. All statistics were performed using GraphPad Prism 6 software. Differences were considered significant when *P* < 0.05.

## RESULTS

### 

#### Study population.

We used a community-based cohort of 40 COPD patients to evaluate antimicrobial immunity at baseline (stable-state) and during virus-associated exacerbation. For baseline analyses of sputum-soluble mediators, samples from all patients were available, with 17 patients (42.5%) classified as frequent exacerbators (clinical characteristics are shown in Table 1). For baseline analyses of sputum cell mRNA [interferons (IFNs) and interferon-stimulated genes (ISGs)], 36 of the 40-patient cohort had sufficient sample for evaluation and, of these, 13 patients (36.1%) were classified as frequent exacerbators (clinical characteristics of this group are shown in Table 1).

**Table 1.** Demographic and clinical characteristics of frequent and infrequent exacerbators included in baseline sputum soluble mediator and mRNA analyses

	Frequent Exacerbators (*n* = 17)	Infrequent Exacerbators (*n* = 23)	*P* Value
*Patients included in baseline sputum-soluble mediator analyses (n = 40)*			
Male sex	12 (70.6%)	16 (69.6%)	1.0
Age, yr	70 (61–79)	70 (63.5–73.8)	0.79
GOLD stage I/II/III/IV	0/9/5/3	8/13/1/1	
Long-term oxygen use	1 (5.8%)	0 (0%)	0.43
Cardiac disease	5 (29.4%)	2 (8.7%)	0.11
Cerebrovascular disease	3 (17.6%)	1 (4.3%)	0.29
Diabetes	2 (11.8%)	1 (4.3%)	0.56
Pulmonary hypertension	0 (0%)	1 (4.3%)	1.0
Inhaled corticosteroid use	12 (70.6%)	9 (39.1%)	0.062
Beta agonist use	16 (94.1%)	19 (82.6%)	0.37
Antimuscarinic inhaler use	15 (88.2%)	14 (60.9%)	0.079
Current smoker	6 (35.5%)	7 (30.4%)	1.0

	Frequent Exacerbators (*n* = 13)	Infrequent Exacerbators (*n* = 23)	*P* Value
*Patients included in baseline mRNA analyses (n = 36)*			
Male sex	10 (76.9%)	16 (69.6%)	0.72
Age, yr	69 (60–74)	67 (59.0–71.5)	0.92
GOLD stage I/II/III/IV	0/8/4/1	8/13/1/1	
Long-term oxygen use	0 (0%)	0 (0%)	1.0
Cardiac disease	3 (23.1%)	2 (8.7%)	0.33
Cerebrovascular disease	3 (23.1%)	1 (4.3%)	0.12
Diabetes	2 (15.38%)	1 (4.3%)	0.54
Pulmonary hypertension	0 (0%)	1 (4.3%)	1.0
Inhaled corticosteroid use	9 (69.2%)	9 (39.1%)	0.16
Beta agonist use	12 (92.3%)	19 (82.6%)	0.63
Antimuscarinic inhaler use	11 (84.6%)	14 (60.9%)	0.26
Current smoker	6 (26.2%)	7 (30.4%)	0.47

GOLD, Global Initiative for Obstructive Lung Disease. Note that only 36 out of 40 subjects had sufficient sample for mRNA analysis.

#### Evaluation of baseline expression of antiviral and proinflammatory mediators.

**Fig. 1.**
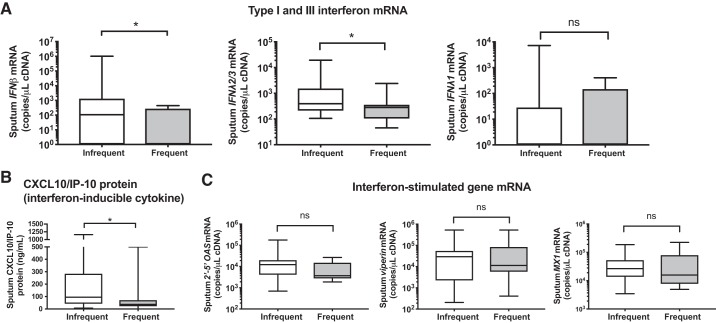
Chronic obstructive pulmonary disease (COPD) patients with frequent exacerbations have reduced antiviral immunity at clinical stability. A cohort of patients with COPD were monitored prospectively and sputum samples were taken when clinically stable for at least 6 wk. Patients were stratified according to exacerbation frequency in the preceding year with patients who experienced ≥2 exacerbation episodes classified as “frequent.” *A*: sputum cell mRNA expression of *IFNβ*, *IFNλ2/3*, and *IFNλ1* was measured by quantitative PCR. *B*: sputum supernatant protein concentrations of CXCL10/IP-10 were measured by ELISA. *C*: sputum cell mRNA expression of interferon-stimulated genes (ISGs). 2′–5′-Oligoadenylate synthetase (*2′–5′ OAS*), *viperin*, and *Mx1* was measured by quantitative PCR. Box-and-whisker plots show median (line within box), interquartile range (IQR; box), and minimum to maximum (whiskers). Statistical comparisons were made using Mann-Whitney *U* test. *P < 0.05; ns, nonsignificant.

**Fig. 2.**
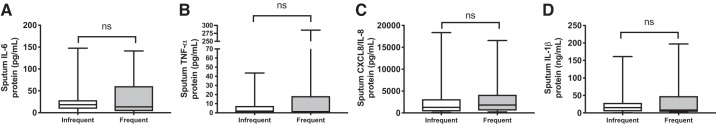
Sputum proinflammatory cytokine concentrations at clinical stability in chronic obstructive pulmonary disease (COPD) subjects stratified according to prior exacerbation frequency. A cohort of patients with COPD were monitored prospectively and sputum samples were taken when clinically stable for at least 6 wk. Patients were stratified according to exacerbation frequency in the preceding year with patients who experienced ≥2 exacerbation episodes classified as “frequent.” Sputum supernatant protein concentrations of IL-6 (*A*), TNF-α (*B*) CXCL8/IL-8 (*C*), and IL-1β (*D*) were measured by ELISA. Data are displayed as box-and-whisker plots showing median (line within box), interquartile range (IQR; box) and minimum to maximum (whiskers). Statistical comparisons were made using Mann-Whitney *U* test; ns, nonsignificant.

Since viruses are a major cause of exacerbations and deficient antiviral immunity has been observed in some, but not all, studies of COPD (1, 17, 20, 22), we hypothesized that frequent exacerbators would have reduced baseline expression of antiviral immune mediators and, thus, an impaired potential to mount protective responses to virus infections. We examined baseline sputum cell mRNA expression of type I and III IFNs and ISGs in 36 patients from the community-based COPD cohort and found that frequent exacerbators had significantly reduced sputum *IFNβ* and *IFNλ2/3* mRNA expression with no difference in *IFNλ1* (Fig. 1*A*). Baseline sputum concentrations of the interferon-inducible protein CXCL10/IP-10 (Fig. 1*B*) were also reduced in frequent exacerbators, but no difference in mRNA expression of other ISGs 2′–5′-oligoadenylate synthetase (*2′–5′ OAS*), *viperin*, or *Mx1* was observed (Fig. 1*C*). There were no differences in baseline sputum levels of proinflammatory cytokines IL-6, TNF-α, CXCL8/IL-8, or IL-1β in frequent versus infrequent exacerbators (Fig. 2, *A*–*D*).

#### Sputum interferon and ISG expression during virus-positive exacerbation.

**Fig. 3.**
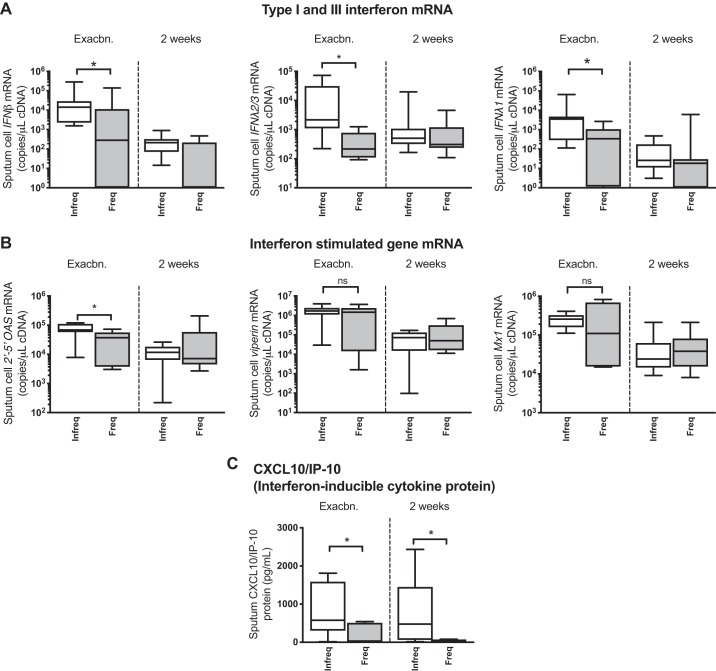
Chronic obstructive pulmonary disease (COPD) patients with frequent exacerbations have reduced antiviral immunity during virus-associated naturally occurring exacerbations. A cohort of patients with COPD were monitored prospectively. Patients were stratified according to exacerbation frequency in the preceding year with patients who experienced ≥2 exacerbation episodes classified as “frequent.” Sputum samples were taken at presentation with exacerbation associated with positive virus detection and 2 wk during exacerbation. Sputum mRNA expression of *IFNβ*, *IFNλ2/3*, and *IFNλ1* (*A*) and 2′–5′-oligoadenylate synthetase (*2′–5′ OAS*), *viperin*, and *Mx1* (*B*) was measured by quantitative PCR. Sputum protein concentrations of CXCL10/IP-10 (*C*) were measured by ELISA. Data are displayed as box-and-whisker plots showing median (line within box), interquartile range (IQR; box), and minimum to maximum (whiskers). Statistical comparisons were made using Mann-Whitney *U* test. *P < 0.05; ns, nonsignificant.

In the community-based cohort, 18 episodes of acute exacerbation with positive virus detection were observed (rhinovirus: *n* = 11, coronavirus: *n* = 4, RSV: *n* = 2, influenza: *n* = 1). Of these, *n* = 7 (38.9%) occurred in patients classified as frequent exacerbators. Clinical characteristics of the exacerbating patients are shown in Table 2. One patient was hospitalized during exacerbation with all other episodes treated in the community. Having observed that frequent exacerbators have reduced capacity to mount an innate antiviral response at stable state, we next examined sputum cell mRNA expression of IFNs and ISGs in the subgroup of patients who developed an exacerbation associated with positive virus detection. Frequent exacerbators had significantly reduced sputum cell expression of *IFNβ*, *IFNλ2/3*, and *IFNλ1* mRNAs at exacerbation presentation (Fig. 3*A*). Sputum *2’-5′OAS* mRNA expression was also significantly reduced at exacerbation presentation in frequent versus infrequent exacerbators with no differences observed for *viperin* or *Mx1* expression (Fig. 3*B*). Frequent exacerbators also had reduced sputum concentrations of the interferon-inducible cytokine CXCL10/IP-10 at exacerbation onset and 2 wk after onset (Fig. 3*C*).

**Table 2.** Characteristics of patients who developed virus-associated exacerbation stratified according to exacerbation frequency

	Frequent Exacerbators (*n* = 7)	Infrequent Exacerbators (*n* = 11)	*P* Value
Age, yr [median (IQR)]	69 (64–74)	71 (52–76)	0.84
Male sex	3 (42.9%)	9 (81.8%)	0.14
GOLD stage (median IQR)	2 (2–3)	2 (1–2)	0.13
Long-term oxygen	0 (0%)	0 (0%)	1.0
Cardiac disease	0 (0%)	1 (9.1%)	1.0
Cerebrovascular disease	1 (14.3%)	0 (0%)	0.39
Diabetes	1 (14.3%)	1 (9.1%)	1.0
Pulmonary hypertension	0 (0%)	0 (0%)	1.0
Inhaled corticosteroid use	4 (57.1%)	3 (27.3%)	0.33
Beta agonist use	6 (85.7%)	7 (63.6%)	0.60
Antimuscarinic inhaler use	4 (57.1%)	6 (54.5%)	1.0
Current smoker	4 (57.1%)	2 (18.2%)	0.14
Treatment during exacerbation			
Antibiotics	5 (71.1%)	3 (27.3%)	0.15
Oral corticosteroids	3 (42.9%)	0 (0%)	0.043

GOLD, Global Initiative for Obstructive Lung Disease; IQR, interquartile range.

#### Ex vivo bronchial epithelial antiviral responses to RV infection are attenuated in frequent exacerbators.

Having observed reduced IFN and ISG expression in frequent exacerbators from in vivo sputum samples taken during exacerbation, we next proceeded to expand upon these findings by determining whether epithelial cells from patients with COPD who are frequent exacerbators have deficient innate responses to RV infection. We used BECs collected bronchoscopically from 16 patients with COPD (*n* = 9 frequent exacerbators, *n* = 7 infrequent exacerbators), differentiated at an air‐liquid interface and infected with RV-A1 at MOI = 0.1. Demographic/clinical characteristics of the included patients are shown in Table 3.

**Table 3.** Baseline characteristics of patients with COPD undergoing bronchoscopic sampling for airway epithelial cell experiments shown in Fig. 5

	Frequent Exacerbators (*n* = 9)	Infrequent Exacerbations (*n* = 7)	*P* Value
Age, yr	77 (72.5–82)	67 (63.5–71)	0.29
GOLD stage			
2	1 (11.1%)	1 (14.3%)	1.0
3	8 (88.9%)	6 (85.7%)	1.0
Male sex	6 (66.7%)	3 (42.9%)	0.61
Inhaled corticosteroid use	7 (77.8%)	3 (42.9%	0.30
Long-acting β-agonist use	7 (77.8%)	3 (42.9%)	0.30
Antimuscarinic inhaler use	7 (77.8%)	3 (42.9%)	0.30
Current smoker	1 (11.1%)	3 (42.9%)	0.26

COPD, chronic obstructive pulmonary disease; GOLD, Global Initiative for Obstructive Lung Disease.

**Fig. 4.**
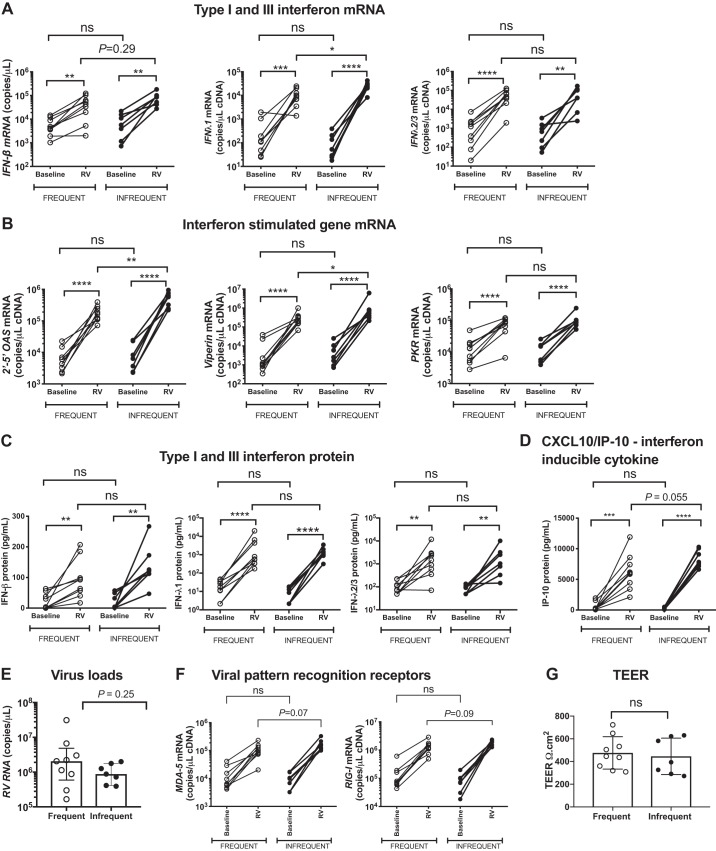
Chronic obstructive pulmonary disease (COPD) patients with frequent exacerbations have deficient ex vivo innate antiviral immune responses to rhinovirus infection in differentiated airway epithelial cells. Primary bronchial epithelial cells (BECs) from 16 patients with Global Initiative for Obstructive Lung Disease (GOLD) stage II or III COPD were differentiated at the air‐liquid interface (ALI) and infected ex vivo with rhinovirus (RV)-A1 or medium control (baseline). Cell lysates and supernatants were harvested postinfection. *A*: *IFNβ*, *IFNλ1*, and *IFNλ2/3*. *B*: 2′–5′-oligoadenylate synthetase (*2′–5′ OAS*), *PKR*, and *viperin* mRNA expression in cell lysates at 72 h was measured by quantitative PCR. IFN-β, IFN-λ1, and IFNλ-2/3 (*C*) and CXCL10/IP-10 (*D*) proteins were measured at 72 h in cell supernatants by ELISA. *E*: cells were harvested at 72 h postinfection and rhinovirus RNA copies were measured by quantitative PCR. *F*: expression of viral pattern recognition receptors *MDA-5* and *RIGI* mRNA was measured by quantitative PCR. *G*: transepithelial electrical resistance (TEER) was measured using an epithelial voltohmmeter with AC current through an electrode set placed in the apical and basal media. An average of three readings was recorded at *day 21* post-ALI when all cultures were fully differentiated before infection. Data represent individual patients and were analyzed by ratio paired *t* test (baseline vs. RV) or Mann-Whitney *U* test (RV frequent exacerbators vs. RV infrequent exacerbator). *P < 0.05, **P < 0.01, ***P < 0.001, ****P < 0.0001; ns, nonsignificant.

Significant induction of *IFNβ* and *IFNλ1* and *IFNλ2/3* mRNAs from baseline was observed in both frequent and infrequent exacerbators at 72 h postinfection (Fig. 4*A*). RV induction of *IFNλ1* mRNA was significantly lower in frequent versus infrequent exacerbators with a similar trend (*P* = 0.29) observed for *IFNβ* mRNA, but no significant difference was observed for *IFNλ2/3* (Fig. 4*A*). RV induction of the ISGs *2’-5′OAS* and *Viperin* mRNA was also significantly lower in frequent versus exacerbators with no significant difference observed for *PKR* (Fig. 4*B*).

Significant induction of IFN-β, IFN-λ1, or IFN-λ2/3 proteins from baseline was also observed in both frequent and infrequent exacerbators at 72 h postinfection (Fig. 4*C*). However, there were no significant differences in the induction of IFN-β, IFN-λ1, or IFN-λ2/3 proteins by RV between frequent and infrequent exacerbators (Fig. 4*C*). Similarly, significant induction of the interferon-inducible cytokine CXCL10/IP-10 from baseline was observed in both groups of subjects with a nonsignificant trend (*P* = 0.055) toward lower induction in frequent exacerbators (Fig. 4*D*). Greater heterogeneity with trend for increased viral load was observed in BECs from frequent exacerbators (Fig. 4*E*). There were also nonsignificant trends toward reduced mRNA expression of the cytosolic viral pattern-recognition receptors MDA-5 and RIG-I following RV infection in frequent versus infrequent exacerbators, suggesting that decreased antiviral immune responses observed in these individuals could be a consequence of decreased recognition of viral invasion (Fig. 4*F*). There was no evidence to suggest barrier function was involved with no difference in transepithelial electrical resistance (TEER) between cells at baseline from frequent exacerbators versus infrequent exacerbators (Fig. 4*G*).

Collectively, these data supported our in vivo findings from exacerbating patients that antiviral responses are inconsistent and reduced in frequent exacerbators and extended these observations by identifying a dysregulated innate immune response in BECs defined by impaired expression of IFN and ISGs.

#### Evaluation of proinflammatory cytokines in RV-infected bronchial epithelial cell cultures.

**Fig. 5.**
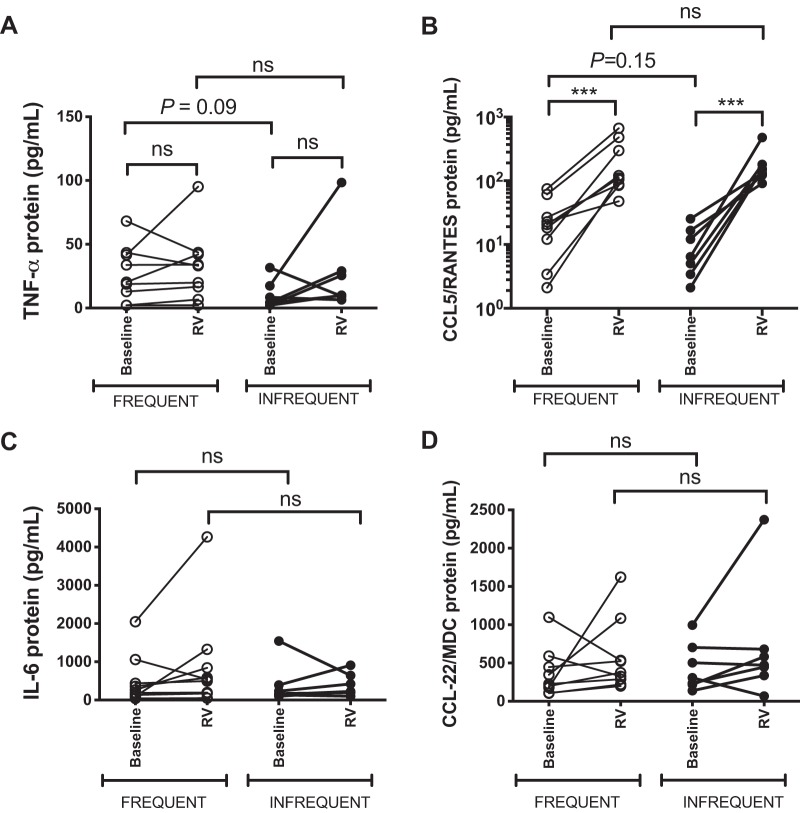
Proinflammatory cytokine concentrations in rhinovirus-infected differentiated airway epithelial cells. Primary bronchial epithelial cells (BECs) from 16 patients with Global Initiative for Obstructive Lung Disease (GOLD) stage II or III chronic obstructive pulmonary disease (COPD) were differentiated at the air‐liquid interface and infected ex vivo with rhinovirus (RV)-A1 or medium control (baseline). Supernatants were harvested at 72 h postinfection. TNF-α (*A*) and CCL5/RANTES (*B*), IL-6 (*C*), and CCL22/MDC (*D*) proteins were measured by ELISA. Data represent individual patients and were analyzed by ratio paired *t* test (baseline vs. RV) or Mann Whitney *U* test (RV frequent exacerbators versus RV infrequent exacerbator). ns, nonsignificant.

Previous in vivo studies have reported that subjects with frequent exacerbations have higher sputum levels of proinflammatory cytokines at stable state (5, 11). Having observed that airway epithelial cells from frequent exacerbators have impaired antiviral immune responses to infection, we next sought to investigate whether this may be related to greater baseline epithelial production of inflammatory cytokines, as there is also some existing evidence that in vitro responses to bacterial infection may be dampened by TNF-α and IL-1β (29). BECs from frequent exacerbators showed nonsignificant trends toward increased TNF-α (*P* = 0.09) and CCL5/RANTES expression (*P* = 0.15) at baseline, with no differences observed for IL-6 or CCL22/MDC (Fig. 5). There were no differences between the level of induction of these cytokines by RV (Fig. 5).

#### Evaluation of sputum bacterial loads at baseline and during virus-associated exacerbation.

**Fig. 6.**
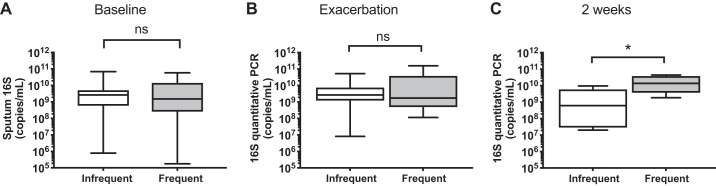
Chronic obstructive pulmonary disease (COPD) patients with frequent exacerbations have increased bacterial loads during virus-associated naturally occurring exacerbations. A cohort of patients with COPD were monitored prospectively. Patients were stratified according to exacerbation frequency in the preceding year with patients who experienced ≥2 exacerbation episodes classified as “frequent.” Sputum samples were taken at presentation with exacerbation associated with positive virus detection and 2 wk during exacerbation. Sputum bacterial loads were measured by 16S quantitative PCR at baseline (*A*), exacerbation (*B*), and 2 wk (*C*). Data are shown as median (interquartile range, IQR) and were analyzed by Mann Whitney *U* test. *P < 0.05. ns, nonsignificant.

Virus-induced exacerbation can trigger subsequent bacterial infection in COPD (13, 21), a secondary phenomenon that is associated with more severe RV infections (21) and inversely related to the interferon responses (33). Given that we observed deficient interferon responses in frequent exacerbators, both in vitro and in vivo, we hypothesized that this phenotype would be associated with increased secondary bacterial infection in exacerbating COPD patients. To investigate this, we measured sputum bacterial loads by 16S quantitative PCR in subjects with COPD at stable state and during virus-associated exacerbation. There were no differences in sputum 16S bacterial loads between frequent and infrequent exacerbators at baseline or exacerbation onset (Fig. 6, *A* and *B*). However, frequent exacerbators had a significant increase (~1 log) in bacterial loads at 2 wk after exacerbation onset (Fig. 6*C*), indicating that this COPD subgroup may have increased likelihood of secondary bacterial infection during virus-induced exacerbation and suggesting that this may be related to a deficient antiviral immune response during exacerbation.

#### No difference in antimicrobial peptide levels between frequent and infrequent exacerbators.

**Fig. 7.**
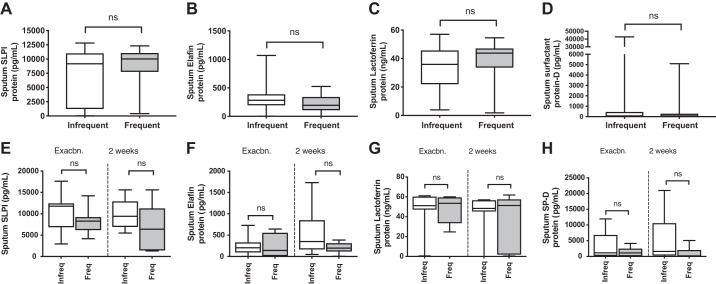
Sputum antimicrobial peptide concentrations at clinical stability and during virus-associated exacerbation in chronic obstructive pulmonary disease (COPD) subjects stratified according to prior exacerbation frequency. A cohort of patients with COPD were monitored prospectively and sputum samples were taken when clinically stable for at least 6 wk and also in a subgroup presenting with virus-associated exacerbation taken at presentation with exacerbation presentation and 2 wk during exacerbation. Patients were stratified according to exacerbation frequency in the preceding year with patients who experienced ≥2 exacerbation episodes classified as “frequent.” Sputum supernatant protein concentrations of secretory leucocyte protease inhibitor (SLPI) (*A*), elafin (*B*), lactoferrin (*C*), and surfactant protein D (*D*) were measured at clinical stability in *n* = 40 subjects by ELISA. Sputum supernatant protein concentrations of SLPI (*E*), elafin (*F*), lactoferrin (*G*), and surfactant protein D (*H*) were measured during exacerbation in *n* = 18 subjects by ELISA. Data are displayed as box and whisker plots showing median (line within box), interquartile range (box), and minimum to maximum (whiskers). Statistical comparisons were made using Mann Whitney *U* test. ns, nonsignificant.

We have previously reported that rhinovirus-induced secondary bacterial infection may occur through neutrophil elastase-mediated cleavage and reduction of the antimicrobial peptides (AMPs) SLPI and elafin, a process that occurs in COPD but not healthy subjects and may be further accentuated by inhaled corticosteroid use (33). Given our observation of increased bacterial loads in frequent exacerbators, we next examined whether this subgroup of COPD patients have concurrently reduced AMP levels. There were no differences in sputum SLPI, elafin, lactoferrin, or surfactant-protein D levels between frequent and infrequent exacerbators either at baseline (Fig. 7, *A*–*D*) or during exacerbation (Fig. 7, *E*–*H*). This suggested that differential expression of these AMPs does not underlie increased secondary bacterial infection in frequent exacerbators.

## DISCUSSION

The underlying mechanisms involved in increased susceptibility to frequent exacerbations remain a crucial research question in COPD. We show here, for the first time, that patients with COPD, who have a history of frequent exacerbations, have reduced airway antimicrobial immunity when assessed at clinical stability and during subsequent virus-associated exacerbation with an associated increase in bacterial loads. We additionally confirm that COPD frequent exacerbators have reduced ex vivo immune responses to RV infection in primary airway epithelial cells, indicating an innate antiviral deficiency. Our data provide an important mechanistic advance in understanding this clinically important COPD subgroup. These abnormalities could predispose such patients to greater risk of acquisition of pathogenic viruses and bacteria and/or promote a greater likelihood of developing an exacerbation following an infection.

Previous studies have shown that ex vivo type I IFN responses to RV infection in bronchoalveolar cells and to influenza infection in BECs are impaired in COPD (17, 22). Patients with COPD also have increased virus loads and enhanced exacerbation severity when experimentally infected with rhinovirus (10, 22). However, defects in antiviral immunity have not been shown universally with other studies reporting the opposite effect of augmented ex vivo antiviral responses in COPD (1, 20). The discrepancy between these COPD studies suggests that defective immunity may not be present in all patients with a number of confounding competing factors, such as disease severity and medications likely to have an influence. It is plausible that patients with frequent exacerbations might represent one subgroup with impaired antiviral immunity. Here, we report that expression of both type I and type III IFNs, *IFNβ*, and *IFNλ2/3* mRNAs is reduced in frequent exacerbators at stable state, suggesting that these patients might have a reduced potential to generate protective responses to virus infection. In support of this hypothesis, we also observed that antiviral responses, including *IFNβ*, *IFNλ1*, and *IFNλ2/3* and the ISGs *2’-5′OAS* and CXCL10/IP-10 were suppressed during exacerbations in frequent exacerbators and that RV induction of *IFNλ1, 2’-5′OAS*, and *Viperin* mRNAs in BECs ex vivo was also reduced in frequent exacerbators.

Our study provides the first evidence that COPD frequent exacerbators have innate antiviral immune dysfunction, suggesting that a reduction in interferon production might underlie an augmented propensity to virus infections and, thus, increased exacerbation frequency observed in these patients. This mechanism is supported by a clinical study, which reported that COPD patients who are frequent exacerbators experience significantly more coryzal episodes than infrequent exacerbators (18). Given that inhaled IFN-β therapy has been shown to confer clinical benefit in a subgroup of severe asthmatic patients who develop a cold (8), our data suggest that such innate immune-boosting therapies could be effective when used in a targeted manner in patients with COPD with evidence of frequent exacerbations and a less effective innate immune response.

Although we observed impaired ex vivo RV induction of IFN and ISG mRNA in frequent exacerbator BECs at 72 h postinfection, we did not observe corresponding differences in protein production of type I and III IFNs by these cells at the same timepoint. Despite a lack of difference in absolute levels of IFN protein by 72 h, the reduced ISG expression observed in frequent exacerbators supports a less effective IFN-induced response in these subjects. Our studies were limited by sample availability only allowing evaluation at a single timepoint.

Bacterial infection is also associated with exacerbation in COPD with increased PCR-based bacterial detection at exacerbation versus stable state, suggesting a causative role (12, 32). Additionally, virus-induced secondary bacterial infection has been reported in both experimental and naturally occurring exacerbations (13, 21). We have previously reported that experimental RV challenge in patients with COPD is associated with increased frequency of secondary bacterial infection compared with healthy subjects (21, 24), an effect that is related to virus loads. Here, we extend these findings to reveal that frequent exacerbators have higher bacterial loads at 2 wk following onset of virus-associated exacerbation, suggesting that this subgroup of COPD patients might be at greatest risk of developing secondary bacterial infection following initial virus infection. These data provide further justification for development of antiviral immunity boosting therapies as an approach to reduce secondary bacterial infection and associated exacerbation severity in COPD.

It is important to note that causation cannot be inferred from the results of our study. Although we found reduced antimicrobial responses in patients with a history of frequent exacerbations, we cannot reliably conclude that these abnormalities directly lead to inherent increased exacerbation risk per se. There are likely to be a number of additional factors that may be contributing to the observed differences and, notably, in stable state analyses, frequent exacerbators showed trends toward greater ICS use and were less commonly observed in GOLD stage 1 group. These confounders could contribute directly or indirectly to the differences observed, as previously reported (33, 37). Therefore, we consider our study to be hypothesis generating and emphasize the importance of future larger in vivo studies to more carefully dissect the influence of various factors and definitively answer the question of whether deficient antimicrobial immunity underlies propensity to exacerbation frequency in COPD.

A further limitation to our study is that the classification of frequent exacerbator was made from clinical history taken at study entry where all patients were asked about exacerbation history in the preceding year. We cannot exclude that recollection of these events may be inaccurate in some subjects. We were unable to corroborate these data with medical records due to the heterogenous nature of locations at which these exacerbations will have been reported and managed (self-management, primary care physician-directed therapy, or hospitalization), preventing a systematic approach to data retrieval. It is also important to note that a relatively short follow-up period was adopted in this study. Since exacerbations may be influenced by seasonal variation in influenza rates, a longer-term follow-up period may offer more accurate information. This short follow-up in our study makes it unsuitable for testing the hypothesis of whether baseline deficiency in antiviral immunity predisposes to subsequent increased frequency of virus-induced exacerbations. An adequately powered study with longer follow-up would be required to formally test this. Our study shows that subjects with prior history of frequent exacerbations have reduced expression of antiviral immune mediators, but it is not a prospective evaluation of this hypothesis. In a study with a much longer follow-up period (median 1,047 days), frequent exacerbators experienced significantly more coryzal episodes than infrequent exacerbators (18), which supports that this phenotype is potentially associated with increased susceptibility to viral infection. Previous studies have also indicated that exacerbation risk in COPD is dynamic and may change from year to year (16, 19), suggesting that factors other than increased clinical attendances (and, thus, potential exposure to viruses) associated with prior frequent exacerbations may govern subsequent exacerbation risk. A longer-term study with repeated airway sampling would be needed to determine whether baseline antimicrobial responses are similarly dynamic in nature.

Identification of an underlying mechanism to explain our observation of impaired antiviral immunity in frequent exacerbators is challenging due to confounding factors, such as inhaled corticosteroid use, making it difficult to determine immune abnormalities that are inherently due to disease phenotype. Previous studies have indicated that frequent exacerbators have increased systemic and local expression of proinflammatory cytokines (5, 11). In the current study, we showed trends toward increased baseline production of inflammatory cytokines TNF-α and CCL5/RANTES, hinting that chronic airway inflammation could contribute to defective antiviral responses upon infection observed in some patients. A previous study in airway epithelial cell cultures demonstrated that proinflammatory cytokine exposure impaired epithelial expression of cathelicidin (29), an antimicrobial peptide that has antibacterial and antiviral properties (4, 7). Exposure to proinflammatory cytokines can induce persistent epigenetic changes that modify innate immune responses. IL-13 exposure of airway epithelial cells from donors with asthma-induced, long-lasting modification of DNA methylation (25). We have previously reported increased RV-induced type I IFN expression in NF-κB p65 heterozygous mice (p65^+/−^) compared with p65^+/+^ mice associated with lower expression of inflammatory COX-2 (2). This is consistent with negative regulation of IFN signaling by inflammatory pathways. Further studies are needed to investigate whether proinflammatory cytokines can similarly impair interferon expression in COPD and the mechanisms underlying this effect.

In conclusion, we show that patients with COPD and a history of frequent exacerbations have reduced antiviral immunity associated with increased secondary bacterial infection. These immune defects may underlie the increased propensity to exacerbations observed in this subgroup and provide evidence to support therapeutic approaches that boost innate immunity in COPD.

## GRANTS

This work was supported by Imperial College Healthcare Trust Biomedical Research Centre Grant P33132; Academy of Medical Sciences and Wellcome Trust Starter Grant; Medical Research Council Program Grant G0600879; British Medical Association H.C. Roscoe Fellowships; BLF/Severin Wunderman Family Foundation Lung Research Program Grant P00/2; Imperial College and NIHR BRC funding scheme; the NIHR Clinical Lecturer funding scheme; Wellcome Trust Clinical Research Training Fellowship WT096382AIA; and British Lung Foundation Pump priming Grant PPRG15-9.

## DISCLOSURES

S. L. Johnston has personally received consultancy fees from Myelo Therapeutics GmbH, Concert Pharmaceuticals, Bayer, and Sanofi Pasteur, and Aviragen; he and his institution received consultancy fees from Synairgen, Novartis, Boehringer Ingelheim, Chiesi, GlaxoSmithKline, and Centocor. S. L. Johnston is an inventor on patents on the use of inhaled interferons for treatment of exacerbations of airway diseases. None of the other authors has any conflicts of interest, financial or otherwise, to disclose.

## AUTHOR CONTRIBUTIONS

A.S., M.C., J.G., J.F., P.A.B.W., C.L.G., S.L.J., N.W.B., and P.M. conceived and designed research; A.S., S.-L.L., M.C., L.J.F., M.-B.T.T., E.B., J.G., P.V., P.S.P., K.S.N., A.R., J.F., N.W.B., and P.M. performed experiments; A.S., S.-L.L., J.G., J.F., N.W.B., and P.M. analyzed data; A.S., S.-L.L., S.L.J., and N.W.B. interpreted results of experiments; A.S. and N.W.B. prepared figures; A.S. drafted manuscript; A.S., S.-L.L., J.G., A.R., S.L.J., N.W.B., and P.M. edited and revised manuscript; A.S., S.-L.L., M.C., L.J.F., M.-B.T.T., E.B., J.G., P.V., P.S.P., K.S.N., A.R., S.L.J., N.W.B., and P.M. approved final version of manuscript.
